# Hydatid Cyst Mimicking a Pericardial Cyst

**Published:** 2017-10

**Authors:** Muzaffer Kahyaoğlu, Çetin Geçmen, İbrahim Akın İzgi

**Affiliations:** *Department of Cardiology, Kartal Koşuyolu Yüksek İhtisas Training and Research Hospital, İstanbul, Turkey.*

**Keywords:** *Echinococcosis*, *Echocardiography*, *Tomography, x-ray, computed*

A 27-year-old female patient presented to our clinic because of atypical chest pain. Her past medical history was unremarkable for any chronic illness. Her vital signs comprised a heart rate of 85 bpm, blood pressure of 125/70 mm Hg, and oxygen saturation of 98%. Physical examination was unremarkable. Electrocardiography showed normal sinus rhythm without any ST-segment and T-wave abnormalities. Her blood work, including electrolytes, complete blood count, hepatic panel, and troponin level, was within the normal limits. Transthoracic echocardiography in the parasternal short-axis view revealed a third chamber besides the left and right ventricles (Figure 1). Modified substernal view showed 2 cysts in the liver (Figure 2). A contrast-enhanced chest computed tomography scan in the axial plane demonstrated 2 cysts in the liver, and there was no pericardial involvement (Figure 3 and Figure 4). The patient was referred for general surgery on the diagnosis of a hydatid cyst.

**Figure 1:**
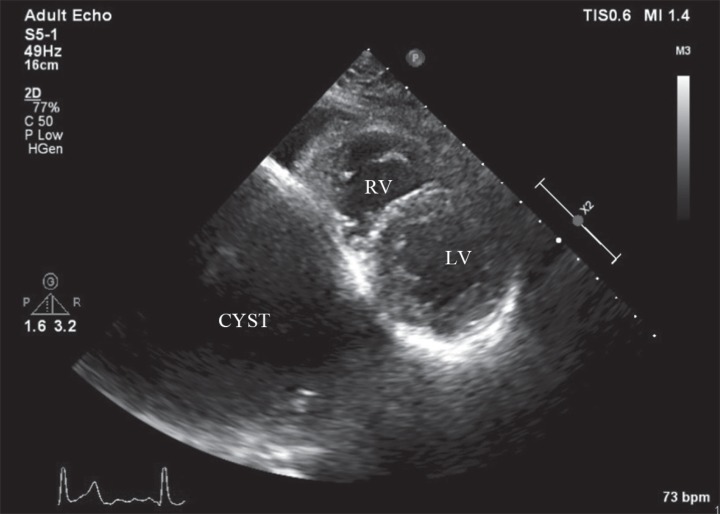
Transthoracic echocardiography in the parasternal short-axis view, showing a third chamber besides the left and right ventricles.

RV, Right ventricle; LV, Left ventricle

**Figure 2:**
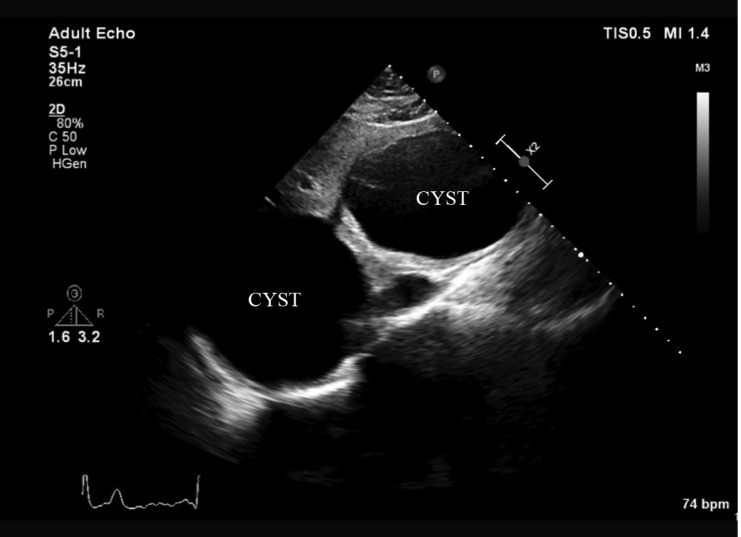
Modified substernal echocardiography view, showing 2 cysts in the liver.

**Figure 3:**
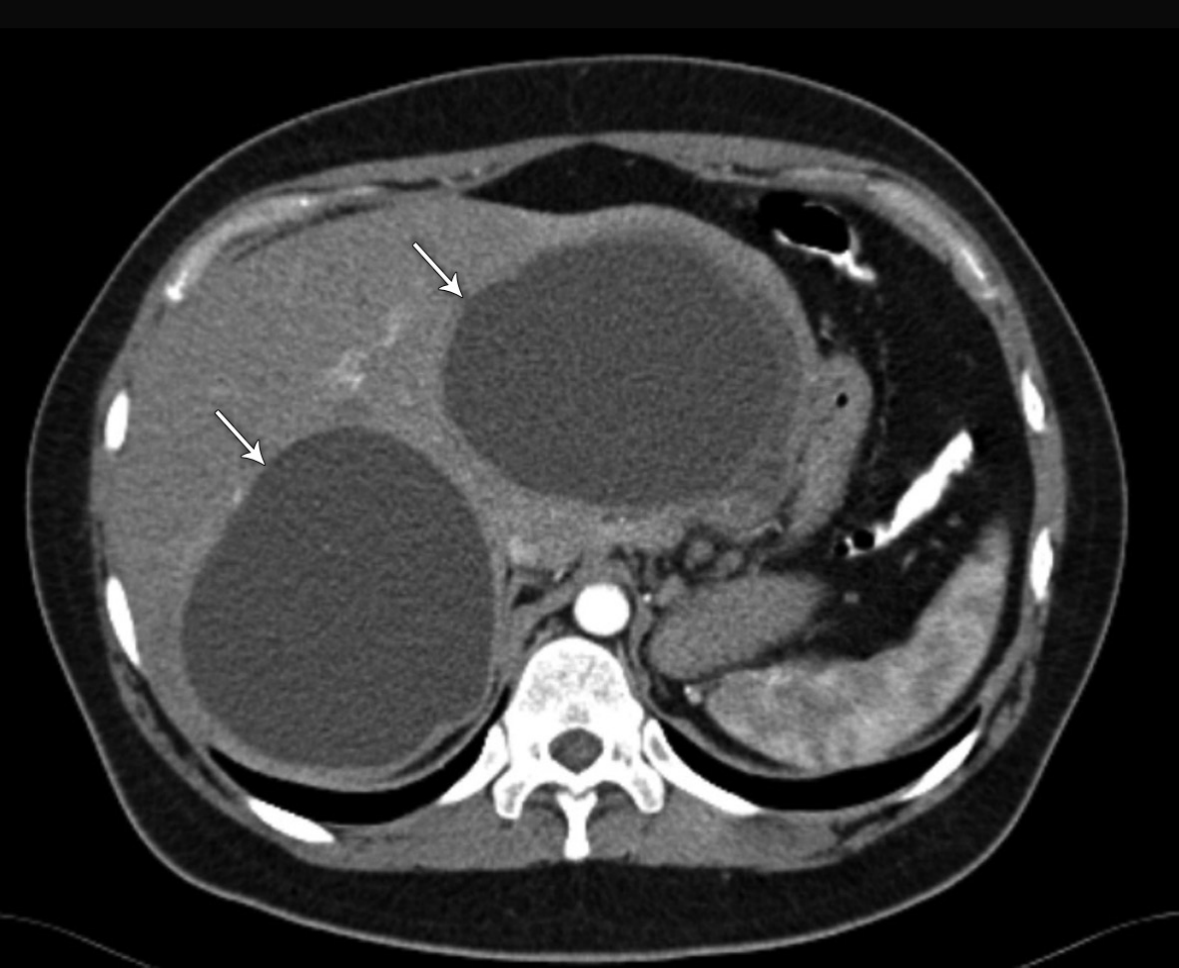
Contrast-enhanced chest computed tomography scan in the axial plane, showing 2 cysts in the liver (arrows).

**Figure 4:**
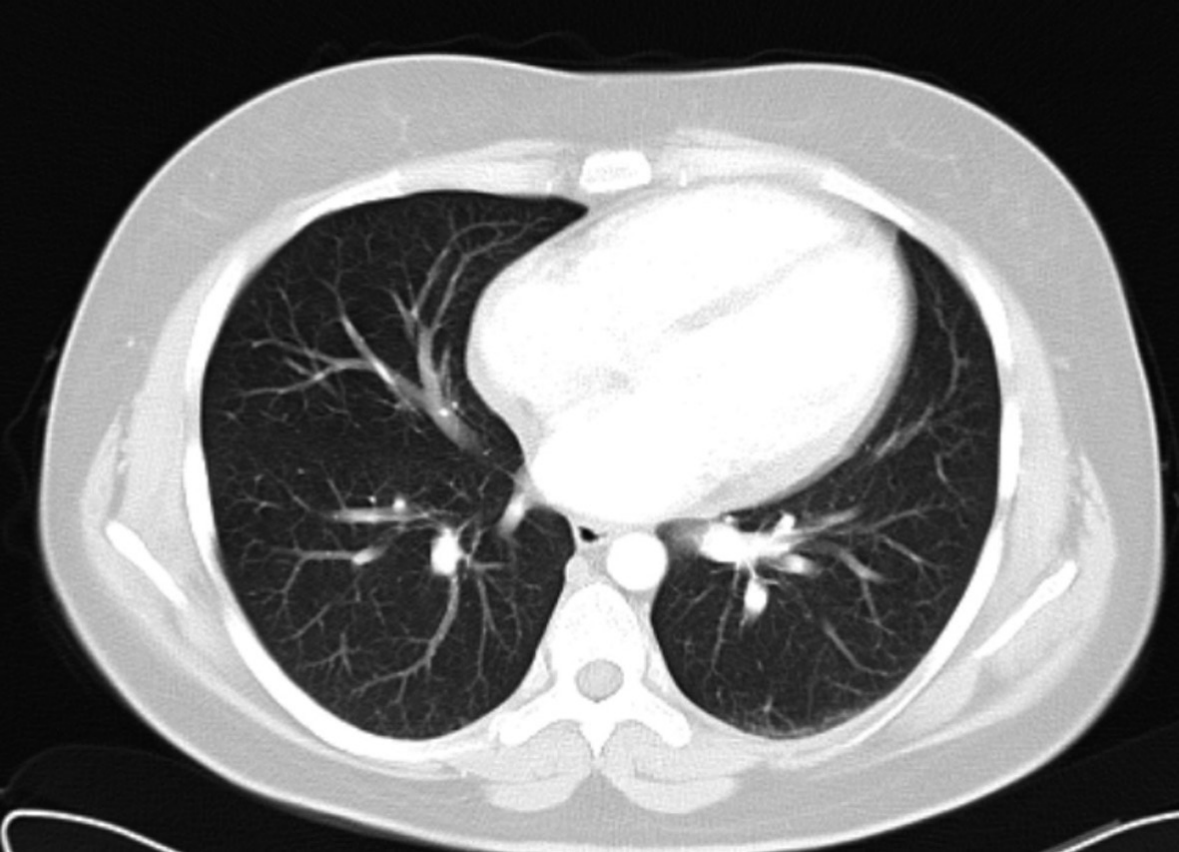
Contrast-enhanced chest computed tomography in the axial plane, showing no pericardial involvement.

## Notes:

***This paper should be cited as:*** Kahyaoğlu M, Geçmen Ç, İzgi İA. Hydatid Cyst Mimicking a Pericardial Cyst. J Teh Univ Heart Ctr 2017;12(4):192-193.

## Notes:

***To watch the following videos, please refer to the relevant URLs:***

Video 1 for Figure 1A.

http://jthc.tums.ac.ir/index.php/jthc/article/view/691/615

Video 2 for Figure 1B.

http://jthc.tums.ac.ir/index.php/jthc/article/view/691/616

